# The role of neutralizing antibodies in prevention of HIV-1 infection: what can we learn from the mother-to-child transmission context?

**DOI:** 10.1186/1742-4690-10-103

**Published:** 2013-10-07

**Authors:** Martine Braibant, Francis Barin

**Affiliations:** 1 Université François-Rabelais, UFR Médecine, Inserm U966 10 bld Tonnellé, cedex, 37032 Tours, France; 2 Centre National de Référence VIH, Laboratoire de Bactériologie-Virologie, CHU Bretonneau, Tours, France

**Keywords:** HIV-1, Neutralizing antibodies, Mother-to-child transmission, Preventive vaccine, Passive immunization, Immunoprophylaxis

## Abstract

In most viral infections, protection through existing vaccines is linked to the presence of vaccine-induced neutralizing antibodies (NAbs). However, more than 30 years after the identification of AIDS, the design of an immunogen able to induce antibodies that would neutralize the highly diverse HIV-1 variants remains one of the most puzzling challenges of the human microbiology. The role of antibodies in protection against HIV-1 can be studied in a natural situation that is the mother-to-child transmission (MTCT) context. Indeed, at least at the end of pregnancy, maternal antibodies of the IgG class are passively transferred to the fetus protecting the neonate from new infections during the first weeks or months of life. During the last few years, strong data, presented in this review, have suggested that some NAbs might confer protection toward neonatal HIV-1 infection. In cases of transmission, it has been shown that the viral population that is transmitted from the mother to the infant is usually homogeneous, genetically restricted and resistant to the maternal HIV-1-specific antibodies. Although the breath of neutralization was not associated with protection, it has not been excluded that NAbs toward specific HIV-1 strains might be associated with a lower rate of MTCT. A better identification of the antibody specificities that could mediate protection toward MTCT of HIV-1 would provide important insights into the antibody responses that would be useful for vaccine development. The most convincing data suggesting that NAbs migh confer protection against HIV-1 infection have been obtained by experiments of passive immunization of newborn macaques with the first generation of human monoclonal broadly neutralizing antibodies (HuMoNAbs). However, these studies, which included only a few selected subtype B challenge viruses, provide data limited to protection against a very restricted number of isolates and therefore have limitations in addressing the hypervariability of HIV-1. The recent identification of highly potent second-generation cross-clade HuMoNAbs provides a new opportunity to evaluate the efficacy of passive immunization to prevent MTCT of HIV-1.

## Review

### Introduction

UNAIDS estimates that there were 34.0 million people living with the human immunodeficiency virus (HIV) at the end of 2011. The development of a safe, effective, preventive HIV vaccine remains among the highest global health priorities. Most vaccines that successfully control viral diseases induce the production of neutralizing antibodies (NAbs) that prevent infection. In the case of HIV-1, a key element for NAbs to be effective in preventing infection is their capacity to neutralize the highly diverse circulating HIV-1 variants, which can differ by more than 30% in the sequences of their envelope glycoproteins. Encouragement for the development of a NAb-based anti-HIV-1 vaccine was provided by successful passive immunization studies in macaque models showing that broadly human monoclonal NAbs administered orally, vaginally or intravenously could prevent the acquisition of infection [1-10]. Despite the fact that in some of these experiments, the viral strain used for challenge was particularly sensitive to neutralization by the passively administered NAbs, these results provided the proof-of-concept that antibodies can block HIV-1 infection. Unfortunately, the capability of vaccine candidates to induce NAbs has turned out to be extremely complex and disappointing. First attempts to develop antibody-based anti-HIV-1 vaccines in man involved recombinant protein immunogens based on monomeric forms of the surface-exposed gp120 component of the envelope glycoprotein (AIDSVAX gp120 B/B and B/E) [11-13]. High levels of antigen-specific antibodies were induced in human vaccinees but failed to neutralize most primary isolates and did not confer protection [14-17]. More recently, the RV144 vaccine trial based on a prime-boost regimen consisting of a recombinant canarypox vector prime (ALVAC-HIV) and gp120 protein boost (AIDSVAX gp120 B/E), showed only moderate protection in low-incidence heterosexuals [18]. These trials, in a real life situation, have indicated the limitations of animal model studies that used only a few selected challenge viral strains. They also highlighted the probable need to develop more sophisticated envelope immunogens. For this, lessons from studies aiming at dissecting the antibody response during natural infection might be particularly useful. It has been shown recently that broadly NAbs, developed after several years of infection by some HIV-1 infected patients, require a high level a somatic mutations to become broad and potent [19-24]. This suggests that an effective vaccine may require a combination of various envelopes to direct B-cell responses through multiple rounds of antibody maturation and mutation process [25]. Another key question for vaccine development is the identification of correlates of protection. Indeed, the specificity and magnitude of the NAbs response required to confer protection against HIV-1 transmission in humans are still unclear, and progress in this field is a key step on the road to an effective HIV-1 vaccine.

### Mother-to-child transmission: a model to identify correlates of protection

**Figure 1:**
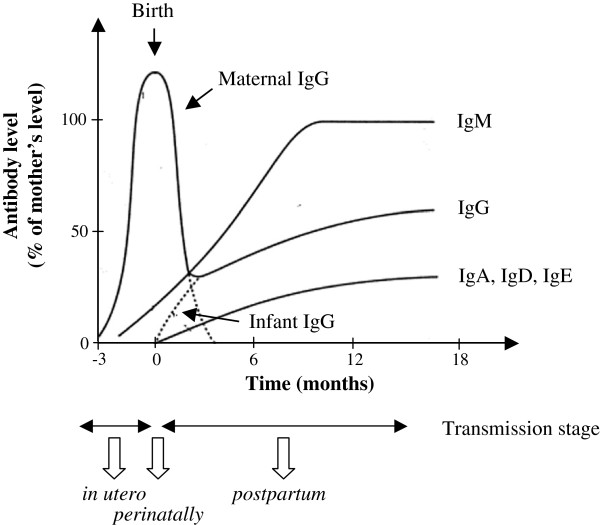
**Infant antibody levels over the three possible stages (*in utero*, *perinatally *or *postpartum*) of mother-to-child transmission of HIV-1.** During pregnancy, maternal IgG are transmitted to the fetus across the placenta, reaching normal or somewhat exceeding adult levels at term. After birth, the IgG transferred from the mother disappear progressively, while the amount of IgG being produced by the infant continues to increase. In contrast, the placenta is relatively impermeable to Ig of other classes, levels of which are therefore very low in the newborn.

Mother-to-child transmission (MTCT) of HIV-1 is currently the principal cause of HIV infections in children. Whereas access of HIV pregnant women to antiretroviral therapy has increased significantly, HIV infection in children remains a major concern. In 2011, an estimated 330,000 children were newly infected with HIV (UNAIDS). Without any antiretroviral treatment, MTCT rate of HIV-1 is around 30 to 40% and occurs mainly at three stages: *in utero* during pregnancy (5-10%), *perinatally* at the time of labor and delivery (15-20%), and *postpartum* through breastfeeding (10-15%) [26-29] (Figure 1). This contrasts with a much lower rate of MTCT of HIV-2 which, in absence of antiretroviral treatment, ranges from 0% to 4% only [30-34]. Despite the high transmission rates of HIV-1, a large number of children exposed to HIV-1 do not become infected. Several maternal factors, including low CD4^+^ T-cells counts and high viral loads are associated with an increasing risk of HIV-1 MTCT [35]. The lower plasma viral load in HIV-2 infected patients, compared to HIV-1 infected patients, may account for the lower MTCT risk for HIV-2 [33,34,36-40].

The role of maternal immune response in limiting HIV-1 transmission to the infant is still unclear. The placenta is relatively impermeable to IgA and IgM, levels of which are therefore very low in the newborn. In contrast, maternal IgG are transported over the placenta by an active process mediated by the FcRn receptor [41]. The timing of maternal IgG passage across the placenta during pregnancy was addressed in several studies (for a review see [42]). During the first trimester, very little IgG is transmitted to the fetus, but in the second trimester, the fetal IgG concentration increases from approximately 10% of the maternal concentration at 17–22 weeks of gestation to 50% at 28–32 weeks [41,43-45]. During the third trimester, fetal IgG levels continue to rise, reaching normal or somewhat exceeding adult levels at term [45-50] (Figure 1). It was illustrated recently in the HIV-1 context in a study that showed that the envelope binding antibody titers were strongly correlated and similar between mothers and their corresponding infants [51]. MTCT constitutes therefore an attractive model to explore the putative protecting role of passively acquired HIV-specific IgG against HIV acquisition. In the absence of data on the putative role of NAbs in prevention of MTCT of HIV-2, this review will focus on HIV-1 infection that has been the subject of intensive investigations.

#### Selective transmission of HIV-1 variants from mothers to infants

**Figure 2:**
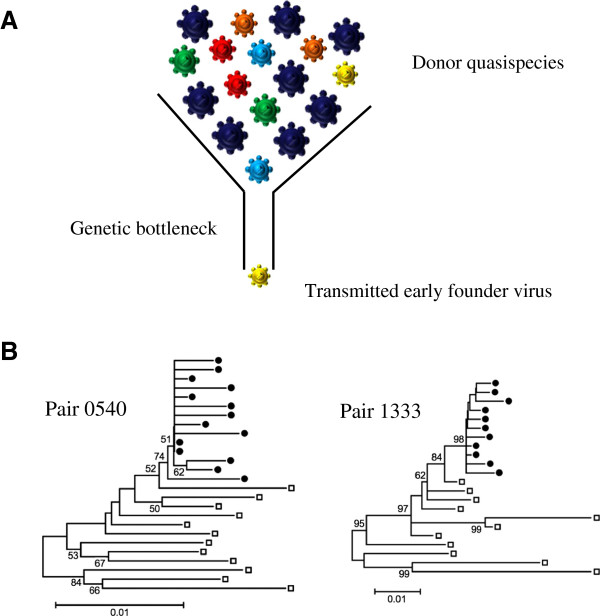
**Selective transmission of HIV-1. A.** The quasispecies of the chronically infected donor is usually composed of a major viral population (dark blue virions), as well as numerous other minor variants. One of these minor variants (yellow virion) successfully crosses the mucosal barrier to generate the infection of the recipient. **B.** The neighbor-joining trees of HIV-1 *env* gp120 nucleotide sequences issued from two mother-infant pairs show the transmission of a single maternal viral variant [85]. Bootstrap values are expressed as percentages per 1000 replicates. Only bootstrap values >50% are indicated. Horizontal branch lengths are drawn to scale, with the black bar denoting 1% divergence. Each symbol denotes a single *env* sequence; **□**, maternal sequence; **●**, infant sequence.

The first molecular studies of *env* sequences diversity issued from infected individuals, adults as well as children, have shown that most of acute/recent infections are characterized by the presence of a highly homogenous genetically-restricted virus population in contrast to the high genetic diversity observed several years later at time of chronic infection [52-62]. These observations led rapidly to the assumption of a substantial bottleneck in virus transmission (Figure 2A). Recently, the use of single genome amplification (SGA) of HIV-1 plasma viral RNA obtained from acutely infected adult individuals, allowed the identification of transmitted/early founder viruses, and the precise estimation of their diversity [63-67]. These studies showed evidence of infection by a single virus in ~80% of heterosexuals and ~60% of HIV-infected men who have sex with men [63,64,66,67]. In contrast, the frequency of multiple variants transmission was found to be higher among intravenous (IV) drug users (~60%), including one subject who was infected by at least 16 variants [65]. The high frequency of mutiple-variants transmission in IV drug users could be due to the absence of a mucosal barrier to virus transmission and higher virus inocula. To date, such large studies have not been conducted on samples from children born to infected mothers. However this question has been considered using different molecular approaches (Table 1) (Figure 2B) [61,68-89]. Data vary from one study to another but all together, comparing the viral population in mother-child pairs, they showed a homogeneous genetically-restricted population in the majority of infected infants (156 of 235; table I), suggesting a selective transmission of some viral variants during MTCT (Figure 2B). The lack of consensus among these studies may be due to the fact that many studies compared only a limited number of mother-child pairs and did not address the route of transmission (*in utero*, *perinatally* or *postpartum* through early breasfeeding). In addition, the ages of the infants at time of sample collection varied considerably among studies.

**Table 1:** Studies of the viral population of HIV-1 infected infants

**Infant samples**			**Infection route**	**Homo-/heterogeneous population ratio**	**References**
**Nature**	Collection time*	**Amplified *env *region**			
PBMCs	2 to 4 months	V3 and V4-V5	Unspecified	3/0	[61]
PBMCs	Unspecified	V3 and V4-V5	Unspecified	4/0	[68]
PBMCs and serum	0 to 4 months	V3	1 IU, 9 unknown	8 (1 IU)/2	[69]
Serum	At birth	V3	1 IU	1/0	[70]
PBMCs	0 to 12.5 months	V3	1 IU, 4 unknown	2/3 (1 IU)	[71]
PBMCs	2 days to 7 weeks	V1-V2-C2	Unspecified	1/2	[72]
PBMCs	1 week to 34 months	V3	Unspecified	7/0	[73]
PBMCs	5 days to 1.5 months	V3	Probably 1 PN, 3 IU	1 (PN)/3 (IU)	[74]
PBMCs and plasma	2 to 40 days	C2-V3	Unspecified	3/1	[75]
PBMCs	1 month	V3	Unspecified	13/4	[76]
Plasma	48 hours or 2 to 6 weeks	V3-V5	9 PN, 14 IU	17 (7 PN, 10 IU)/6 (2 PN, 4 IU)	[77]
PBMCs	0 or 2 to 6 months	C2-V4	3 PN or early PP, 1 IU	3 (2 PN, 1 IU)/1	[78]
PBMCs	0 or 6 weeks	V1-V4	7 PN or early PP, 6 IU	6 (2 IU, 4 PN)/7 (4 IU, 3 PN)	[79]
PBMCs	48 hours or 2 to 6 weeks	V3-V5	7 PN, 14 IU	15 (5 PN, 10 IU)/6 (2 PN, 4 IU)	[80]
PBMCs or plasma	6 weeks	V1-V5	8 PN or early PP, 1 IU, 3 BF	11/1 (PN)	[81]
PBMCs or cord blood	0 or 6 to 15 months	V1-V3	3 PP, 1 IU	4/0	[82]
PBMCs	4 to 18 months	V1-V5	Unspecified	0/3	[83]
Plasma	0 or 6 weeks	V1-V2	23 PN, 25 IU	20 (6 PN, 14 IU)/28 (17 PN, 11 IU)	[84]
Plasma	48 hours or 6 weeks	V1-V5	11 PN, 6 IU	14 (9 PN, 5 IU)/3 (2 PN, 1 IU)	[85]
PBMCs	2 to 4 months	V1-V5	6 PN or early PP	5/1	[86]
Plasma	30 to 66 days	V1-V5	5 PN	3/2	[87]
Plasma	0 or 6 weeks	V1-V5	9 PN or early PP, 10 IU	13 (5 PN, 8 IU)/6 (4 PN, 2 IU)	[88]
Plasma	3 or 6 months	complete *env*	2 PP	2/0	[89]
				total: 156/235	

* Time of the first PCR or coculture positive collected sample after birth.

PN: *perinatally*; IU: *in utero*; PP: *postpartum* through breastfeeding.

Focusing on transmission through breastfeeding, three recent studies have shown that there is no or very limited viral compartmentalization between milk and blood, suggesting that breast milk viruses are typical of circulating viruses [90-92]. As with other routes of MTCT, a genetic bottleneck that restrict the number of variants transmitted through breastfeeding to a single or a small number of variants was reported [82].

Several studies examined the molecular characteristics of the envelope glycoproteins of transmitted viruses that might explain the selective process. Two studies of Wolinky’s group, in which MTCT route was not defined, reported that the potential N-linked glycosylation site (PNGS) proximal to the first cysteine of the V3 loop (N295) was strikingly absent from the infant’s sequence populations but present in the majority of the maternal sequence sets [61,68]. This observation has not been found by other investigators [69,70,73,74]. Studies performed on sequences encoding the full-length gp120 reported shorter variable regions and/or fewer PNGS in clade A and C viruses transmitted from mother-to-infant, mainly perinatally or early postpartum [81,86,88]. In contrast, we and others did not find altered gp120 length or PNGS number in the clade B and CRF01-AE viruses transmitted perinatally from mothers to their infants [85,87]. Interestingly enough, similar observations were reported during or shortly after heterosexual transmission of HIV-1. Transmitted viruses of subtype A and C showed a more compact and less glycosylated gp120 but this molecular property was not observed for sexually transmitted viruses of clade B [55,93,94]. This highlighted potential differences in the biology of the different subtypes, regardless of their transmission mode. Although we did not find shorter gp120s or fewer PNGS in maternally transmitted CRF01-AE viruses, we however found a limited number of PNGS, N301 in V3 and N386 in C3, that seemed to be conserved in all infected infants but were not uniformly present in their mothers, suggesting that they may confer an advantage on the virus to be transmitted [85]. By comparing functional properties of pseudotyped viruses expressing envelopes carrying or not N301 and/or N386, we confirmed that these two PNGS may play a role in resistance to autologous sera [95]. Interestingly, the N-linked glycan at position 301 was recently shown to be important for HIV-1 neutralization by several new broad and potent monoclonal NAbs of the PGT series (PGT125-128, PGT130 and PGT131) [96]. Similarly, Moore *et al.* recently reported in two HIV-1-infected adults the apparition of the N-linked glycan at position 332 targeted by PGT128, through immune escape from earlier strain-specific antibodies [97]. It could be possible that NAbs drive viral selection during MTCT, leading to variants with conserved glycan motifs that would confer a selective advantage.

#### Neutralizing antibodies: in search of correlates of protection in the MTCT context

MTCT offers a unique opportunity to explore the putative role of NAbs in restricting or preventing infection, especially when the transmission occurs in presence of high levels of passively acquired maternal IgG, i.e. during the *perinatal* and early breastfeeding periods. Conflicting results have been obtained concerning the role of maternal NAbs in reducing MTCT of HIV-1. Early studies, each relatively small, showed that non-transmitting mothers had more frequently detected and/or higher titers of autologous NAbs than transmitting mothers, suggesting a role for maternal NAbs in reducing MTCT [98-101]. Supporting this model, a few studies have suggested that transmitted viruses are escape variants resistant to neutralization by maternal antibodies [80-82,102,103]. However, other studies did not confirm these findings [88,89,95,104-106]. The observed discordant results may be due to small sample sizes, disparate maternal and infant sample collection time points, and a lack of identification of the route of transmission in several studies (*in utero*, in absence or in presence of only low levels of IgG, *versus perinatally* or early *postpartum,* in presence of high levels of IgG).

Because transmitted variants were found to be resistant to neutralization by their own mother’s plasma in several studies, we previously hypothesized that protective antibodies might be those with a broad neutralizing activity. To test this hypothesis, we conducted three large studies, i.e. two studies in Thailand [107,108], and one study in French patients [109], in which we compared the breadth and levels of NAbs in sera of transmitting and non-transmitting mothers, using panels of heterologous primary isolates of different clades. Our data did not support an association between the breadth of HIV-1 neutralizing activity and a lower rate of MTCT of HIV-1, whether transmission occurred *in utero* or *perinatally* (the infants were not breast-fed). Similar results were obtained by others in Kenyan infants of HIV-1 positive mothers [110]. All these data clearly indicate that infants who acquired broad and potent NAbs able to neutralize heterologous HIV-1 variants of different subtypes from their mothers did not show a reduced risk of infection. However, our studies suggested that particular HIV-1 variants might be indicators of NAbs associated with *in vivo* protection. Indeed we found that NAbs toward a CRF01-AE isolate, MBA, were significantly associated with a lower rate of MTCT in Thailand. Similarly, statistically significant higher frequencies or titers of NAbs toward several strains were observed in non-transmitting mothers in our French study when the clade B-infected mothers or non clade-B-infected mothers were analyzed separately [109]. Collectively, these data suggest that particular HIV-1 variants may provide a measure of protective antibodies depending of the population. These HIV-1 variants may be particular strains on which epitopes targeted by protective NAbs would be particularly well exposed in contrast to those of non-protective antibodies. The identification of these strains would be dependent of the HIV-1 subtype of the studied population suggesting that the neutralizing response specificities might vary between viral subtypes. Conducting large studies on homogeneous populations (in terms of geographical origin, viral clade, maternal viral loads and antiretroviral prophylaxis) to limit confounding viral and/or host factors would be necessary to identify such indicator strains.

**Figure 3:**
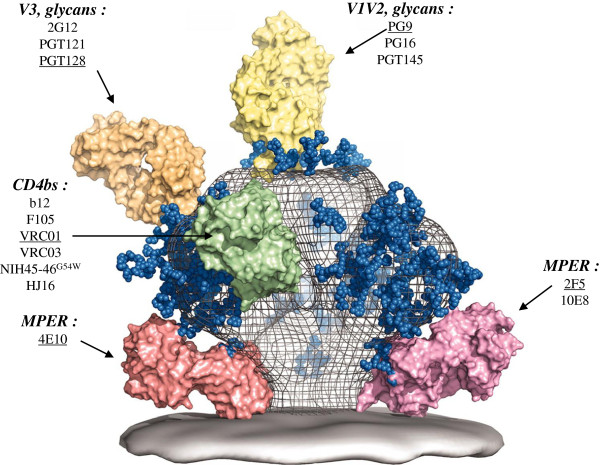
**A model of the HIV-1 Env spike with selected HuMoNAbs Fabs bound to their conserved epitopes.** Adapted with permission from AAAS - Burton *et al.*[118]. The names of selected HuMoNAbs are underlined. The locations of their targeted epitopes are indicated in bold and italic. The name of other HuMoNabs targeting similar epitopes is included [96,115-117,152,153].

The targeted epitope(s) of the neutralizing response associated with protection was not explored in studies cited above. To date, only one recent study conducted in a cohort of South African patients addressed this question by focusing on the putative role of maternal gp41-specific antibodies passively transferred to newborns [111]. An association of antibodies to the membrane-proximal external region (MPER) of gp41 with virus neutralization and protection was reported. This observation needs to be confirmed by other studies. More particularly it should be extended to other important conserved epitopes using various techniques, such as serum absorption with recombinant Env proteins or functional neutralization assay with various Env-pseudotyped viruses, that have been recently developed to dissect the neutralizing activity present in human sera [112-114]. These epitopes, most of them being of conformational nature, were identified thanks to the isolation of human monoclonal broadly neutralizing antibodies (HuMoNAbs) [96,115-118] (Figure 3). They are conserved regions of the HIV-1 envelope glycoprotein such as the CD4 binding site (CD4bs) on gp120, quaternary structure dependent epitopes on the V1-V2 variable loops of gp120, glycan-dependent epitopes involving the V3 region of gp120, and the MPER of gp41. A fine comparison of the specificity of the neutralizing response between transmitting and non-transmitting mothers by such extensive serum mapping using Env proteins or Env-pseudotyped viruses derived from HIV-1 strains indicator of protective antibodies should help to define more precisely if some antibody specificities correlate with protection in the MTCT context.

In the absence of antiretroviral prophylaxis, HIV is transmitted via breastfeeding to only 10-15% of infants, despite daily milk exposure. This suggests that breast milk may contain antiviral immune factors protecting most infants from mucosal HIV infections. HIV-1 envelope-specific antibody responses have been detected in milk. However, despite a predominance of secretory IgA in milk, the magnitude of the HIV-1 envelope-specific IgA activity is lower than that of envelope-specific IgG activity [119-122]. Similarly, the predominant SIV envelope-specific antibodies in milk from lactating rhesus monkeys are of the IgG isotype [123]. This is in accordance with what has been observed in other mucosal compartments, such as genital tract and saliva [119,120,124-128]. A few studies explored the putative role of envelope-specific antibodies present in breast milk of infected mothers to prevent infant infection. Quantitative studies of HIV or SIV envelope-specific binding antibodies did not reveal any difference between transmitting and non-transmitting mothers [129-131]. However, the neutralizing activity of these antibodies was not analyzed in these studies. Two recent studies made extensive comparisons of the neutralizing and antibody-dependent cellular cytotoxicity (ADCC) responses in breast milk and plasma from African HIV-infected lactating women [122,132]. Although lower in magnitude, the neutralizing and ADCC activities in milk were directly correlated with those in plasma and were primarily mediated by plasma-derived IgG antibodies. Similarly, SIV-specific IgA antibodies had limited neutralization potency compared to SIV-specific IgG in SIV-infected rhesus monkeys [123]. This suggests that IgA-mediated neutralizing and ADCC responses did not play a major role in preventing MTCT transmission via breastfeeding. However, the capacity of milk IgA to block HIV-1 transcytosis across epithelial cells was not studied. Such a protective role, recently reported for preventing vaginal and rectal infections in animal models, cannot be excluded [133,134]. The preventing role of IgG responses in mothers’ milk is difficult to explore due to the presence in newborns of high physiological levels of passively acquired maternal IgG. One study reported a higher magnitude of milk IgG-mediated ADCC in HIV-1-infected women who did not transmit HIV to their infants postnatally than in transmitters [132]. This suggests that an efficient vaccine should be able to induce a protective humoral response that could be transferred also through breastfeeding.

### Studies of passive immunization in newborn macaques: sterilizing immunity

**Figure 4:**
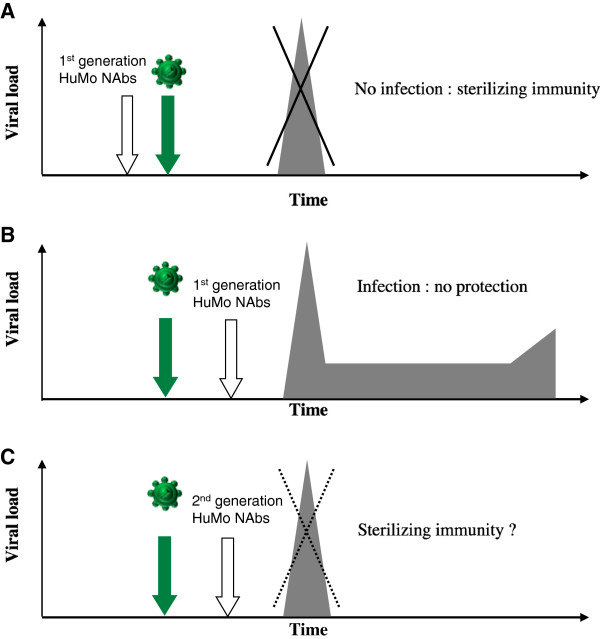
**Studies of passive immunization in newborn macaques. A.** Passive administration of high concentrations of various combinations of the first-generation HuMoNAbs (b12, 2G12, 2F5, 4E10, F105) (white arrow) before or simultaneously with intravenous or oral challenge with SHIVs (green arrow) protected neonatal rhesus macaques against infection: there was no infection [4,149,150]. **B.** There was no protection when the first-generation HuMoNAbs (white arrow) were administered more than 12 hours post-virus inoculation (green arrow) [151]. **C.** New second-generation HuMoNAbs (PG-, PGT-, VRC-series) that are 10- to 100-fold more potent *in vitro* than the first-generation HuMoNAbs have been identified [96,115-117,152,153]. It would be interesting to re-evaluate the potential protective potency of NAbs in newborn macaques when administered either before or after (white arrow) viral exposure (green arrow).

The development of MTCT models of SIV infection in macaques has been very useful to evaluate the potential of passively transferred antibodies to prevent infection. A first attempt of passive immunization of newborn rhesus macaques with pooled sera from chronically SIV-infected rhesus macaques has been shown to be protective from oral SIV exposure that mimics the mucosal exposure occurring during perinatal and breast-milk HIV-1 transmission (Table 2) [1]. However, the precise mechanism for the protection could not be established as no neutralizing activity of these pooled sera could be detected *in vitro* against the challenge virus. At about the same time, the first HuMoNAbs, F105, 2F5, 4E10, 2G12 and b12, were generated and characterized (Figure 3). F105 and b12 are directed against the CD4 binding site of gp120 [135,136], 2G12 recognizes a conformational carbohydrate-dependent epitope on gp120 [137], and 2F5 and 4E10 are directed against the MPER of gp41 [138,139]. To evaluate the capacity of these HuMoNAbs to prevent HIV-1 infection, chimeric simian-human immunodeficiency viruses (SHIV) were used. Because the SIV and HIV envelope glycoproteins are too divergent to analyze their sensitivity to NAbs, SHIV were constructed based on a SIV backbone in which the SIV *env* gene was replaced by an HIV-1 *env* gene. The first SHIV constructs expressed the *env* gene of the T-cell line laboratory-adapted (TCLA) HIV strain IIIB which is highly neutralization-sensitive [140-143], but constructs using *env* genes from primary HIV-1 isolates were subsequently made, generating more pathogenic and more neutralization-resistant SHIV viruses [144-147]. Using the SHIV-macaque model, several studies have shown that passive administration of high concentrations of various combinations of the first-generation HuMoNAbs protected neonatal rhesus macaques against high-dose intravenous or oral challenge with SHIVs (Table 2) [3,4,6,148-151]. These results provided the proof-of-concept that antibodies can prevent infection. However the current SHIV models, which include only a few HIV Envs, provide data that are limited to protection against a restricted number of isolates and therefore has limitations when considering the high diversity of HIV-1 isolates. In addition, these studies highlighted the importance of the timing of administration, since HuMoNAbs must be present at the time of viral challenge or only a few hours later to prevent the establishment of infection (sterilizing immunity) of the newborn macaques (Table 2) (Figure 4A). There was no protection when antibodies were administered more than 12 hours post-virus inoculation (Figure 4B). Recently, the association of several technological advances allowed the identification of new second-generation HuMoNAbs (particularly the PG, PGT and VRC series) that are tenfold to 100-fold more potent *in vitro* than the first-generation HuMoNAbs used in passive immunization studies [96,115-117,152,153] (Figure 3). One of these antibodies, PGT121, was able to mediate sterilizing immunity against high-dose mucosal viral challenge in adult rhesus macaques at serum concentrations that were significantly lower than those observed in previous studies [154]. In addition, Klein *et al.* re-examined the possibility to use antibody transfer as a therapeutic modality [155]. Using a humanized mice model and combinations of these new NAbs that target complementary sites on the HIV-1 spike protein, they showed that these antibodies could effectively control HIV-1 infection and suppress viral load to levels below detection. Given these new data, it would be interesting to evaluate the protective potency of these new NAbs in newborn macaques for both pre-exposure and post-exposure prophylaxies (Figure 4C). Indeed, a recent study of passive administration of neutralizing IgG (including the HuMoNAb b12) at levels insufficient to block infection to newborn macaques before oral challenge with a SHIV virus has shown that immunized macaques rapidly developed NAbs and had a significantly reduced plasma viremia [156]. This supports the use of NAbs to augment B cell responses and viral control in perinatal settings, although further studies are needed to understand the mechanisms underlying their beneficial effects.

**Table 2:** Studies of passive immunization in newborn macaques

**Treatment**		**Challenge**		**Sterile protection**	**References**
**Antibody (Route, Concentration)**	**Infusion timing**	**Virus**	**Route**		
SIV hyperimmune serum (SC, 20 mL/kg)	- Postnatal 2 days before challenge	SIVmac 251 (10^5^ TCID50)	Oral	- 2/2	[1]
	- Postnatal 2 days before challenge and 5 and 12 days after challenge			- 4/4	
	- Postanatal 3 weeks after challenge			- 0/3	
- HIV immune globin (HIVIG) (IV, 400 mg/kg)	Postnatal 24 hours before challenge	SHIV89.6PD (40 TCID50)	IV	- 0/3	[3]
- 2F5 (IV, 15 mg/kg)				- 0/3	
- 2G12 (IV, 15 mg/kg)				- 0/3	
- 2F5 / 2G12 (IV, 15 mg/kg of each)				- 0/3	
- HIVIG/2F5/2G12 (IV, 400 mg/kg of HIVIG, 15 mg/kg of each HuMoNAb)				- 3/6	
F105/2G12/2F5 (IV, 10 mg/kg of each)	Pre- and postnatal 1–4 hours before and 8 days after challenge	SHIVIIIB-vpu^+^ (10 AID50)	Oral	4/4	[4]
2G12/b12/2 F5 (IV, 10 mg/kg of each)	Postnatal 1 hour before and 8 days after challenge	- SHIVIIIB-vpu^+^ (10 AID50)	Oral	- 2/2	[148]
		- SHIV-89.6P (15 AID50)		- 1/4	
F105/2G12/2F5 (IV, 10 mg/kg of each)	- Postnatal 3–4 hours before challenge and 8 days after challenge	SHIVIIIB-vpu^+^ (10 AID50)	Oral	- 2/2	[149]
	- Postnatal 1 hour and 8 days after challenge			- 2/2	
2G12/b12/2F5/4E10 (IV, 30 mg/kg of each except 4E10 at 11.5 mg/kg)	Postnatal 1 hour and 8 days after challenge	SHIV-89.6P (15 AID50)	Oral	2/4	[6]
2G12/2F5/4E10 (IM, 40 mg/kg of each)	Postnatal 1 hour and 8 days after challenge	SHIV-89.6P (15 AID50)	Oral	4/4	[150]
- 2G12/b12/2 F5/4E10 (IV, 30 mg/kg of each)	- Postnatal 1 hour and 8 days after challenge	SHIV-89.6P (15 AID50)	Oral	- 3/4	[151]
	- Post natal 12 hours and 8 days after challenge			- 1/4	
- 2G12/2F5/4E10 (IM, 40 mg/kg of each)	- Postnatal 24 hours and 9 days post challenge			- 0/4	

TCID50: 50% tissue culture infectious dose; AID50: 50% animal infectious dose.

SC: subcutaneous; IV: intravenous; IM: intramuscular.

### Prevention of mother to child transmission : immunoprophylaxis

MTCT of HIV-1 infection remains a significant problem in developing countries. Antiretroviral (ARV) prophylaxis can reduce the number of infections, but it does not eliminate the transmission risk. ARV efficacy is highly dependent on strict adherence to daily administration that is difficult to achieve for many women/babies. Even with optimal prophylaxis, infections occur at a rate of 1 to 5% at 6 months of age in infants of HIV-1 infected mothers who breastfeed [157]. Additional interventions that ideally do not rely on daily adherence to prevent transmission during prolonged breastfeeding need to be identified [158]. The use of an anti-HIV-1 passive immunization approach in addition to ARV prophylaxis could provide additional protection and deserves to be explored.

Passive immunization experiments of rhesus macaques have proven that NAbs can protect from MTCT. In humans, there are only 2 phase III studies of passive immunization to prevent MTCT that have been conducted. Both used polyclonal hyperimmune globulin preparations from HIV-1-infected donors. The first study was conducted in 1993–1997 in the United States in a non-breastfeeding population of HIV-1 infected pregnant women receiving zidovudine prophylaxis [159]. The second was conducted in 2004–2006 in Uganda in breastfeeding pregnant mothers receiving single-dose nevirapine [160]. Although the infusion of HIV hyperimmune preparations was proven to be safe, no additional benefit of these polyclonal preparations compared to antiretroviral treatment alone was shown in these two studies. However, it may be possible that polyclonal preparations did not contain enough NAbs specificities able to provide sterilizing immunity. The use of HuMoNAbs may be more appropriate to reach this goal and it was proposed to use them in human clinical trials [161,162]. However, several of the first-generation antibodies (b12, 2F5 and 2G12) were found ineffective or only partly effective against non-B viruses, particularly toward a panel of clade C Env-pseudotyped viruses derived from primary isolates of South African infected children and consequently did not seem to be relevant to prevent MTCT in populations where non-B viruses predominate [163]. In contrast, the second-generation of broadly HuMoNAbs directed against quaternary epitopes (PG9, PG16), the CD4bs epitopes (VRC01, NIH45-46^G54W^) and V3 glycan-dependent epitopes (PGT121, PGT128) were recently found to be able to neutralize most of HIV-1 non-B variants transmitted through breastfeeding [89,164-166]. Similarly, by comparing functional properties of CRF01-AE variants transmitted *perinatally* to infants with those of their chronically infected mothers, we recently found that all the transmitted variants were highly sensitive to both PG9 and PG16, significantly more sensitive than the maternal variants [95]. Together, these data would suggest that this new generation of HuMoNAbs could be efficient in passive immunization strategies. HIV prevention trials in African breastfed infants using the antibody HuMoNAb VRC01 are currently considered [167].

## Conclusions

There are strong evidences for a selective advantage of the HIV-1 variants that are transmitted from the mother to the infant in the presence of maternal HIV-1-specific antibodies. An association between presence or titers of NAbs toward different HIV-1 strains and a lower rate of mother-to-child transmission has been found in several studies, suggesting that some NAbs could contribute to protection toward neonatal infection. Together, these data suggest that some neutralizing specificities might be protective and, in case of transmission, that the transmitted variants escape these specificities. Dissecting the antibody specificities that mediate protection toward MTCT of HIV-1 could provide important clues for identification of correlates of protection that would be useful for vaccine development. Experiments of passive immunization in newborn macaques have shown that first-generation HuMoNAbs can fully protect against SHIV infection, providing additional proof that NAbs can inhibit MTCT. The recent identification of highly potent second-generation HuMoNAbs provides a new opportunity to evaluate the efficacy of passive immunization to prevent mother-to-child transmission of HIV-1.

## Abbreviations

NAbs: Neutralizing antibodies; MTCT: Mother-to-child transmission; HuMoNAbs: Human monoclonal neutralizing antibodies; HIV: Human immunodeficiency virus; PNGS: Potential N-linked glycosylation site; MPER: Membrane-proximal external region; CD4bs: CD4 binding site; ADCC: Antibody-dependent cellular cytotoxicity; SHIV: Simian-human immunodeficiency virus; TCLA virus: T-cell line laboratory-adapted virus; ARV: Antiretroviral.

## Competing interests

The authors declare no competing interests.

## Authors’ contributions

MB and FB wrote the manuscript. All authors read and approve the final manuscript.
